# Structural Characterization of the Highly Restricted Down Syndrome Critical Region on 21q22.13: New *KCNJ6* and *DSCR4* Transcript Isoforms

**DOI:** 10.3389/fgene.2021.770359

**Published:** 2021-12-08

**Authors:** Francesca Antonaros, Margherita Pitocco, Domenico Abete, Beatrice Vione, Allison Piovesan, Lorenza Vitale, Pierluigi Strippoli, Maria Caracausi, Maria Chiara Pelleri

**Affiliations:** Unit of Histology, Embryology and Applied Biology, Department of Experimental, Diagnostic and Specialty Medicine (DIMES), University of Bologna, Bologna, Italy

**Keywords:** Down syndrome, trisomy 21 (Down syndrome), HR-DSCR, *KCNJ6*, *DSCR4*, RNA isoforms

## Abstract

Down syndrome (DS) is caused by trisomy of chromosome 21 and it is the most common genetic cause of intellectual disability (ID) in humans. Subjects with DS show a typical phenotype marked by facial dysmorphisms and ID. Partial trisomy 21 (PT21) is a rare genotype characterized by the duplication of a delimited chromosome 21 (Hsa21) portion and it may or may not be associated with DS diagnosis. The highly restricted Down syndrome critical region (HR-DSCR) is a region of Hsa21 present in three copies in all individuals with PT21 and a diagnosis of DS. This region, located on distal 21q22.13, is 34 kbp long and does not include characterized genes. The HR-DSCR is annotated as an intergenic region between *KCNJ6-201* transcript encoding for potassium inwardly rectifying channel subfamily J member 6 and *DSCR4-201* transcript encoding Down syndrome critical region 4. Two transcripts recently identified by massive RNA-sequencing (RNA-Seq) and automatically annotated on Ensembl database reveal that the HR-DSCR seems to be partially crossed by *KCNJ6-202* and *DSCR4-202* isoforms. *KCNJ6-202* shares the coding sequence with *KCNJ6-201* which is involved in many physiological processes, including heart rate in cardiac cells and circuit activity in neuronal cells. *DSCR4-202* transcript has the first two exons in common with *DSCR4-201*, the only experimentally verified gene uniquely present in *Hominidae*. In this study, we performed *in silico* and *in vitro* analyses of the HR-DSCR. Bioinformatic data, obtained using Sequence Read Archive (SRA) and SRA-BLAST software, were confirmed by Reverse Transcription-Polymerase Chain Reaction (RT-PCR) and Sanger sequencing on a panel of human tissues. Our data demonstrate that the HR-DSCR cannot be defined as an intergenic region. Further studies are needed to investigate the functional role of the new transcripts, likely involved in DS phenotypes.

## 1 Introduction

Human chromosome 21 (Hsa21) is the smallest human autosome, and it represents 1–1.5% of the human genome (Piovesan et al., 2019). Jérôme Lejeune was the first to associate Down syndrome (DS) to trisomy 21 (T21) (Lejeune et al., 1959) leading to an increasing interest about Hsa21 sequence. After chromosome 22, Hsa21 was the second human chromosome whose sequence was determined (Hattori et al., 2000). To date, Hsa21 is the human chromosome with the lowest number of genes (Piovesan et al., 2016); it has 228 known protein-coding and 106 non-coding RNA (ncRNA) genes (http://www.ncbi.nlm.nih.gov/gene). However, it is still unclear how the specific alterations of Hsa21 genes are linked to the phenotypic features of subjects with DS.

The study of the extremely rare cases of partial trisomy 21 (PT21) is a key model for linking genotype and phenotype in subjects with DS (Korbel et al., 2009; Lyle et al., 2009; Pelleri et al., 2016; Pelleri et al., 2017). PT21 is characterized by the duplication of only a segment of Hsa21 that may or may not be associated with DS diagnosis. Since the early ‘70s, numerous attempts have been made to understand which segments of Hsa21 present in triplicate are responsible for the phenotype of DS. Briefly, after the development of chromosomal banding techniques, many articles have described the presence of 3 G regions on the distal half of the long arm (21q22) of Hsa21 in PT21 subjects with DS phenotype (Caspersson et al., 1970). At the same time, the absence of the syndrome was reported in PT21 subjects where the short arm (21p) and the proximal part of the long arm (21q21) were present in three copies (Shapiro, 1999). These results indicate a relationship between the 21q22 region and DS phenotype, while 21p and 21q21 resulted not essential for the development of DS pathogenesis (Gustavson, 1964; Hamerton, 1971; Daniel, 1979; Eggermann and Schwanitz, 2011). In 1974, Niebuhr and coll. reviewed 14 previously described cases of PT21 subjects and identified a region of 17.4 Mb in 21q22 *locus* that is needed to manifest DS (Niebuhr, 1974). The limits of 17.4 Mb region were further restricted thanks to the analysis of other PT21 cases (McCormick et al., 1989; Rahmani et al., 1989; Delabar et al., 1993) that allowed the identification of the Down syndrome critical region (DSCR) (Rahmani et al., 1989). Systematic attempts were then published in 2009 to identify “critical regions” on Hsa21 for several distinct phenotypes observed in DS (Korbel et al., 2009; Lyle et al., 2009). A systematic reanalysis of all described cases of PT21 from 1973 to 2019 was performed (Pelleri et al., 2016; Pelleri et al., 2019) rather than trying to identify subregions responsible for distinct phenotypes, we focused on the diagnosis of DS implying its most common clinical findings: a recognizable form of intellectual disability (ID) and typical DS *facies*. It was demonstrated that the presence of a highly restricted Down syndrome critical region (HR-DSCR) of only 34 kb located on distal 21q22.13 from 37,929,229 to 37,963,130 shared by all subjects with PT21 and with diagnosis of DS (Pelleri et al., 2019).

**FIGURE 1:**
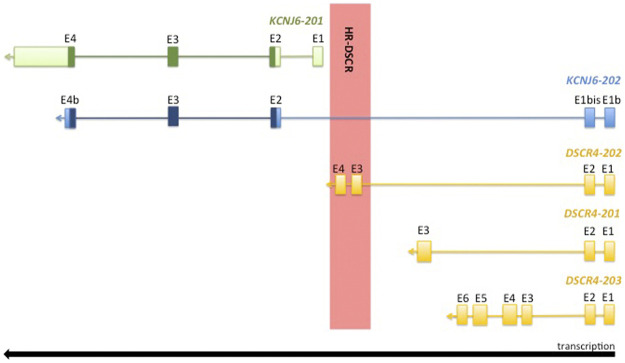
Graphical representation of 21q22.13 *locus*. The coding sequence is highlighted in dark green and dark blue. The exon coordinates of the analysed transcripts are reported in Table 1.

The HR-DSCR sequence was conserved during evolution in the *Hominidae* family, suggesting a role in the development of higher brain function (Saber et al., 2016). The HR-DSCR is annotated as an intergenic region between *KCNJ6-201* gene encoding for potassium inwardly rectifying channel subfamily J member 6 and *DSCR4-201* gene encoding Down syndrome critical region 4 (Figure 1).

The HR-DSCR has not yet been characterized, but recently, two transcripts partially crossing this region were identified by massive RNA sequencing (RNA-Seq) and automatically annotated in Ensembl Genome Browser. The first transcript is *KCNJ6-202* (ENST00000645093.1) which extends the *KCNJ6* *locus* through the addition of two exons upstream of HR-DSCR and includes this region within a long intron (see Figure 1). *KCNJ6-201* (NM_002240.5) and *KCNJ6-202* are both transcripted from the reverse DNA strand and they share the complete coding sequence (CCDS) (CCDS42927) (highlighted in dark green and dark blue in Figure 1) encoding for a G protein-activated inward rectifier potassium channel 2 (GIRK2; NP_002231.1). In humans, *GIRK2* is predominantly expressed in central nervous system (CNS) neurons and generates slow inhibitory post-synaptic potentials playing an important role in the control of resting membrane potential and regulation of cellular excitability (Cooper et al., 2012). *KCNJ6-201* mutation is associated with Keppen-Lubinsky Syndrome **(**OMIM #614098), a rare condition characterized by severe developmental delay, facial dysmorphism, and ID.

*KCNJ6-202* partially shares its two additional exons with *DSCR4* isoforms (see genomic coordinates reported in Table 1), and together with *DSCR4-202* is the only transcript that overlaps HR-DSCR (see Figure 1).

**TABLE 1:** Exon coordinates of the analysed transcripts. The exons inside the HR-DSCR *locus* are reported in red. The exons encompassing the HR-DSCR are reported in green. The graphical representation of the transcripts is shown in Figure 1. Genomic coordinates refer to the Genome Reference Consortium (GRC) human genome assembly GRCh38, or hg38.

	Transcripts				
	*KCNJ6-201*	*KCNJ6-202*	*DSCR4-201*	*DSCR4-202*	*DSCR4-203*
Exon coordinates	E1: 37,961,457-37,915,884	E1b: 38,121,345-38,121,128	E1: 38,121,360-38,121,128	E1: 38,121,360-38,121,128	E1: 38,121,360-38,121,128
	E2: 37,840,709-37,840,658	E1bis: 38,120,408-38,120,307	E2: 38,120,408-38,120,307	E2: 38,120,408-38,120,307	E2: 38,120,408-38,120,307
	E3: 37,715,131-37,714,211	E2: 37,840,709-37,840,658	E3: 38,054,773-38,054,011	E3: 37,953,005-37,952,821	E3: 38,099,338-38,099,277
	E4: 37,625,484-37,607,373	E3: 37,715,131-37,714,211	—	E4: 37,951,702-37,951,425	E4: 38,097,509-38,097,267
	—	E4b: 37,625,484-37,623,559	—	—	E5: 38,094,840-38,094,758
	—	—	—	—	E6: 38,093,348-38,093,244

*DSCR4,* also known as *DCRB*, is expressed predominantly in the placenta and its function is still unknown (Dunn et al., 2006). Three isoforms, *DSCR4-201* (ENST00000398948), *DSCR4-202* (ENST00000398948.5), and *DSCR4-203* (ENST00000482032.1) are annotated in the genome databases and they share the first two exons and the promoter. The first exon is separated by 92-bp sequence from *DSCR8* gene; this separator sequence is a bidirectional promoter that starts the transcription of both genes. *DSCR4-202* is the only transcript that overlaps HR-DSCR with two exons (see Figure 1). *DSCR4* gene is the only annotated gene specific of the *Hominidae* family, which includes humans and great apes, recognized for unique complex social behaviour and intellectual abilities (Saber et al., 2016).

The aim of this work is to characterize HR-DSCR *locus* looking for genetic determinants that might justify the correlation between this region and the main phenotypic characteristics of DS. Using Sequence Read Archive-Basic Local Alignment Search Tool (SRA-BLAST) software we performed an *in silico* analysis to report *KCNJ6* and *DSCR4* expression values in several tissues. In the same panel of tissues, *KCNJ6* and *DSCR4* expression values were detected by Reverse Transcription-Polymerase Chain Reaction (RT-PCR).

## 2 Materials and Methods

### 2.1 HR-DSCR Computational Analysis

Ensembl Genome Browser (https://www.ensembl.org/index.html) was used to annotate the exon limits of the investigated isoforms and to observe their location in comparison to the HR-DSCR limits. BLASTN software was used to align exon junction sequences (used as query sequence) with RNA-Seq experiments selected as explained below, in order to evaluate tissue isoform expression. For reads obtained by the bio-project “Illumina HiSeq 2500 of 4 human trisomy 21 and 4 human normal control blood cells” (PRJNA635385), recently annotated on Gene, we used local SRA-BLAST (https://trace.ncbi.nlm.nih.gov/Traces/sra/sra.cgi?view=software) to perform the alignments. We considered only alignments with at least 95% of query cover and 97% of identity as significative.

### 2.2 RNA-Seq Experiment Selection

RNA-Seq experiments were selected on *KCNJ6* (https://www.ncbi.nlm.nih.gov/gene/3763) and *DSCR4* (https://www.ncbi.nlm.nih.gov/gene/10281) NCBI Gene schedule under the “Expression” heading. The three bio-projects selected were: “HPA RNA-seq normal tissues (PRJEB4337)”; “RNA sequencing of total RNA from 20 human tissues (PRJNA280600)”; “Illumina bodyMap2 transcriptome (PRJEB2445)”; we excluded “Tissue-specific circular RNA induction during human foetal development (PRJNA270632)” because it included experiments performed on circular RNA during the fetal phase. We included the bio-project “Illumina HiSeq 2500 of 4 human trisomy 21 and 4 human normal control blood cells” (PRJNA635385), in which RNA-Seq was performed on RNA samples extracted following the same procedures and conditions of RNA used in this work for *in vitro* analyses (Antonaros et al., 2021b).

We considered RNA samples obtained by tissues (both euploid and T21) involved in the DS phenotype and which were available in our laboratory to confirm computational data with molecular biology experiments.

The euploid tissues selected from the four bio-projects were: adrenal gland, brain, cerebellum, cerebral cortex, heart, liver, placenta, skeletal muscle, skin, testis, thymus, thyroid, white blood cells and blood cells. The T21 tissue selected was T21 blood cells (see Supplementary Table S1).

### 2.3 RNA Sample Selection for *in Vitro* Analyses

For the molecular biology experiments, we used commercially available RNA samples (Clontech, Mountain View, CA), derived from the following human euploid tissues: adrenal gland, brain, cerebellum, cerebral cortex, heart, liver, placenta, skeletal muscle, testis, thymus, thyroid. The main characteristics of RNA donors are listed in Supplementary Data Set S1. The results reported by the *in vitro* analyses were compared with those reported by the RNA-Seq experiments carried out on the same tissues and listed above.

It was possible to detect the expression of *KCNJ6* and *DSCR4* both in euploid and trisomic tissues only for RNA derived from fibroblast and blood cells obtained as follows.

Normal and T21 blood samples were collected at the Neonatology Unit of S. Orsola-Malpighi Hospital of Bologna, in the context of the “Genotype-phenotype correlation in trisomy 21 (Down syndrome)” project (for ethical approval, see “Declarations” section below). Blood samples were collected in ethylenediaminetetraacetic acid (EDTA)-coated blood collection tubes, and plasma fraction was isolated within 2 h from blood collection according to our previously published work (Antonaros et al., 2021a). Five mL of denaturing solution were added to the remaining blood fraction and it was stored at -20°C until RNA extraction. The main features of donors are listed in Supplementary Data Set S1. The results reported by the *in vitro* analyses were compared with those reported by the RNA-Seq experiments (PRJNA635385) performed on normal and T21 blood samples.

Normal and T21 primary fibroblast cell lines were provided by Galliera Genetic Bank (GGB), member of the “Network Telethon of Genetic Biobanks.” All the cell lines were tested for *mycoplasma* to exclude possible contamination. Furthermore, a karyotype analysis was carried out on T21 cell lines by GGB to confirm the cytogenetic diagnosis. Five mL of denaturing solution was added to the cell flasks and they were stored at −20°C until RNA extraction. The main features of donors are listed in Supplementary Data Set S1. The results reported by the *in vitro* analyses performed on euploid fibrobast cell lines were compared with those reported by the RNA-Seq experiments (PRJEB4337) performed on normal skin tissues.

RNA extraction from blood samples and fibroblast cell lines was performed with the method of Chomczynski and Sacchi (Chomczynski and Sacchi, 1987), and RNA quantity and quality were verified through electrophoresis on agarose gel (visualization and quantification with GelDoc 2000 and Quantity One software, Bio-Rad Laboratories, Hercules, CA, United States) and through Nanodrop spectrophotometer (ND-1000 spectrophotometer, Thermo Fisher Scientific, Waltham, MA, United States).

### 2.4 Primer Design

Primer pairs were designed with “Amplify 3” program (Engels, 1993) following the standard criteria described by Sharrocks (Sharrocks, 1994). Briefly, primers should have an annealing temperature (T_a_) between 55°C and 65°C and the difference between the two temperatures should be less than 2°C. Guanines (Gs) and Cytosines (Cs) content should be around 40–60% and it is recommended to avoid secondary structures such as hairpins, self-dimers and cross-dimers which could be caused by self-complementarity of forward and reverse primers. The primers must have biological specificity to avoid non-specific amplifications within the genome (Ye et al., 2012). Primer BLAST software (https://www.ncbi.nlm.nih.gov/tools/primer-blast/) was used to evaluate the biological specificity by comparing the primer pair sequences versus the entire collection of nucleotides (nr/nt). The primer sequences and characteristics were reported in Supplementary Table S2.

### 2.5 *In vitro* Expression Analysis: RT-PCR and Sanger Sequencing

Pools of RNA samples were created from RNA derived from T21 and normal control blood cells: one with three T21 blood cell RNA samples (330 ng of each) and the second with three normal control blood cell RNA samples (330 ng of each) (see Supplementary Data Set S1). Pools of RNA samples were created from RNA from T21 and normal control fibroblasts: one with three T21 fibroblast RNA samples (330 ng of each) and the second with three normal control fibroblast RNA samples (330 ng of each) (see Supplementary Data Set S1).

Reverse transcription (RT) was conducted on 1 µg of RNA (in a total volume of 20 µL). Complementary DNA (cDNA) was obtained using “SuperScript III First-strand Synthesis Supermix” kit (Invitrogen by Life technologies, Grand Island, NY, United States) according to manufacturer instructions: 10 min (minutes) at 25°C, 30 min at 50°C, 5 min at 85°C. cDNA obtained from RT was amplify through PCR to detect *KCNJ6* and *DSCR4* expression. Each 25 µL PCR reaction contained 2.5 µL of cDNA, 1 unit (U) of Platinum Taq DNA Polymerase (Invitrogen; Thermo Fisher Scientific), 2.5 µL of PCR buffer (10x), 25 mM of MgCl2 (final 1.5 mM), 5 mM dNTPs mix (final 0.2 mM), 0.2 µM of each primer. The mixture was denatured at 94°C for 5 min and the PCR was performed for 45 cycles under the following conditions: denaturation at 94°C for 30 seconds (s), annealing at 58–62°C (depending on primer features) for 30 s or 1 min (30 s for transcripts <1,000 bp, 1 min for transcripts >1,000 bp), and extension at 72°C for 1 min. The final extension cycle of 72°C was for 10 min. RT reactions were performed using MJ Research PTC-200 Thermal Cycler and PCR reactions were performed using GenePro TC-E-48D Thermal Cycler.

Amplification products were analyzed by electrophoresis on agarose gel and by Sanger sequencing. The RT-PCR products were purified with GenElute PCR Clean-up Kit (Sigma-Aldrich, Saint Louis, MO, United States) and prepared for Sanger sequencing. The mixture was prepared following BigDye Kit protocol (Applied Biosystems, Carlsbad, CA, United States): 10 ng of amplicon, 0.32 µM of forward or reverse primer, 2 µL of PCR buffer, 0.5–1 µL of BigDye (0.5 µL for transcripts <500 bp, 1 µL for transcripts ≥ 500 bp), and sterile water up to 10 µL of final volume. Automated sequencing was performed with Applied Biosystems ABI 3730 DNA.

Finally, we compared the results obtained by RT-PCR, SRA-BLAST alignments and data available on Ensembl to determine the structure of *KCNJ6* and *DSCR4* transcripts.

## 3 Results

### 3.1 HR-DSCR Computational Analysis

Figure 1 shows a graphical representation of *KCNJ6-201* and *KCNJ6-202* isoforms and Table 1 reports the exon coordinates of each transcript. The first and second exons of *KCNJ6-202* are present only in this isoform and, in this work, they are named as Exon (E)1b and E1bis (see Figure 1 and Table 1). The third and the fourth exons of *KCNJ6-202* correspond to the second and third exons of *KCNJ6-201* (named as E2 and E3 in both isoforms; see Figure 1 and Table 1). The fifth exon of *KCNJ6-202* (called E4b; see Figure 1 and Table 1) is identical, for the first part of the sequence, to the fourth exon of *KCNJ6-201* (called E4; see Figure 1 and Table 1). *KCNJ6-202* transcript includes the HR-DSCR in a long intron located between the second and third exons (E1bis and E2; see Figure 1 and Table 1).

Regarding *DSCR4* gene isoforms to date, *DSCR4-201*, *DSCR4-202* and *DSCR4-203* are annotated in the genome databases. The three isoforms share the first and second exons of which the first is similar to the first exon of *KCNJ6-202* (E1b; see Figure 1 and Table 1), and the second exon is totally shared with *KCNJ6-202* (E1bis; see Figure 1 and Table 1). *DSCR4-201*, the only *DSCR4* isoform registered on National Center for Biotechnology Information (NCBI) Gene database, was discovered in 1997 via Expressed Sequence Tag (EST) (Nakamura et al., 1997) and it has three exons (see Figure 1 and Table 1). *DSCR4-202* transcript is described as long non-coding-RNA (lncRNA)*,* and it has 4 exons of which the third and the fourth are included in the HR-DSCR (see Figure 1 and Table 1). The longest isoform of *DSCR4* gene is *DSCR4-203*, which has 6 exons (see Figure 1 and Table 1) and it is described as lncRNA.

Finally, BLASTN software was used to align exon junction sequences (used as query sequence) with RNA-Seq experiments. The exon junction sequence of 50 bp was used as query sequence, and it was generated by the last 25 nucleotides (nt) of the previous exon (e.g., exon 1) and the first 25 nt of the next exon (e.g., exon 2). *KCNJ6-201* and *KCNJ6-202* shared 2 exon junctions, one generated by the last 25 nt of the E2 and the first 25 nt of the E3, and the second generated by the last 25 nt of the E3 and the first 25 nt of the E4 (see Figure 1 and Table 1). *KCNJ6-202* and the three isoforms of *DSCR4* shared 1 exon junction generated by the last 25 nt of the E1 (or E1b) and the first 25 nt of the E2 (or E1bis) (see Figure 1 and Table 1).

### 3.2 RNA-Seq Experiment Selection

We analyzed 86 RNA-Seq experiments selected following the parameters mentioned above (Material and Methods Section 2.3). Exon junction sequences of each transcript were listed in the respective Supplementary Tables: *KCNJ6-201* (see Supplementary Table S3); *KCNJ6-202* (see Supplementary Table S4); *DSCR4-201* (see Supplementary Table S5)*; DSCR4-202* (see Supplementary Table S6)*; DSCR4-203* (see Supplementary Table S7) were compared to RNA-Seq experiments using SRA-BLAST.

#### 3.2.1 Sequence Alignment Between RNA Experiments and *KCNJ6-201* Transcript

The reads generated by RNA-Seq experiments were aligned with the exon junctions of *KCNJ6-201* transcript. The alignments predict the expression of the entire *KCNJ6-201* transcript in cerebral tissues (brain, cerebellum and cerebral cortex) confirming the data reported in literature (Fagerberg et al., 2014), and predict the expression of part of the transcript in adrenal gland and testis (see Table 2). For more details see Supplementary Table S3.

**TABLE 2:** Summary results of *KCNJ6-201* expression by SRA-BLAST analysis. The results of alignments between the query sequences derived from *KCNJ6-201* and the reads generated by RNA-Seq experiments were reported in this table for each tissue. Only alignments with at least 95% of query cover and 97% of identity were considered as significant and reported in the table.

*KCNJ6-201*			
Tissue	Expression		
	E1-E2	E2-E3	E3-E4
Adrenal Gland	No	3 alignments	No
Brain	14 alignments	25 alignments	13 alignments
Cerebellum	17 alignments	15 alignments	9 alignments
Cerebral cortex	9 alignments	12 alignments	10 alignments
Heart	No	No	No
Liver	No	No	No
Placenta	No	No	No
Skeletal muscle	No	No	No
Skin	No	No	No
Testis	6 alignments	2 alignments	No
Thymus	No	No	No
Thyroid	No	No	No
White blood cells	No	No	No
T21 blood cells	No	No	No
Normal control blood cells	No	No	No

#### 3.2.2 Sequence Alignment Between RNA Experiments and *KCNJ6-202* Transcript

The reads generated by RNA-Seq experiments were aligned with exon junctions of *KCNJ6-202* transcript and no alignments with the entire isoform were reported. The first exon junction (E1b-E1bis), detected in placenta and testis, is the same of *DSCR4-201*, *DSCR4-202,* and *DSCR4-203* and was indicated with an asterisk (*) in Tables 3–6 *.* E1bis-E2, which is exclusive of this transcript, did not generate alignments with RNA-Seq experiments. The last part of the transcript (E2-E4), being in common with *KCNJ6* ***-*** *201* isoform, generated the same alignments reported in Table 2 in the columns “E2-E3” and “E3-E4” (see Tables 2, 3). For more details see Supplementary Table S4).

**TABLE 3:** Summary results of *KCNJ6-202* expression by SRA-BLAST analysis. The results of alignments between the query sequences derived from *KCNJ6-202* and the reads generated by RNA-Seq experiments were reported in this table for each tissue. Only alignments with at least 95% of query cover and 97% of identity were considered as significant and reported in the table.

*KCNJ6-202*				
Tissue	Expression			
	E1b-E1bis	E1bis-E2	E2-E3	E3-E4
Adrenal Gland	No	No	3 alignments	No
Brain	No	No	25 alignments	13 alignments
Cerebellum	No	No	15 alignments	9 alignments
Cerebral cortex	No	No	12 alignments	10 alignments
Heart	No	No	No	No
Liver	No	No	No	No
Placenta	17 alignments*	No	No	No
Skeletal muscle	No	No	No	No
Skin	No	No	No	No
Testis	33 alignments*	No	2 alignments	No
Thymus	No	No	No	No
Thyroid	No	No	No	No
White blood cells	No	No	No	No
T21 blood cells	No	No	No	No
Normal control blood cells	No	No	No	No

^*^The first exon junction (E1b-E1bis), detected in placenta and testis, is the same of the E1-E2 exon junction found in the *DSCR4* isoforms.

**TABLE 4:** Summary results of *DSCR4-201* expression by SRA-BLAST analysis. The results of alignments between the query sequences derived from *DSCR4-201* and the reads generated by RNA-Seq experiments were reported in this table for each tissue. Only alignments with at least 95% of query cover and 97% of identity were considered as significant and reported in the table.

*DSCR4-201*		
Tissue	Expression	
	E1-E2	E2-E3
Adrenal Gland	No	No
Brain	No	No
Cerebellum	No	No
Cerebral cortex	No	No
Heart	No	No
Liver	No	No
Placenta	17 alignments*	10 alignments
Skeletal muscle	No	No
Skin	No	No
Testis	33 alignments*	2 alignments
Thymus	No	No
Thyroid	No	No
White blood cells	No	No
T21 blood cells	No	No
Normal control blood cells	No	No

^*^The first exon junction (E1-E2), detected in placenta and testis, is the same of the first exon junctions found in *KCNJ6-202* and in the other *DSCR4* isoforms.

**TABLE 5:** Summary results of *DSCR4-202* expression by SRA-BLAST analysis. The results of alignments between the query sequences derived from *DSCR4-202* and the reads generated by RNA-Seq experiments were reported in this table for each tissue. Only alignments with at least 95% of query cover and 97% of identity were considered as significant and reported in the table.

*DSCR4-202*			
Tissue	Expression		
	E1-E2	E2-E3	E3-E4
Adrenal Gland	No	No	No
Brain	No	No	No
Cerebellum	No	No	No
Cerebral cortex	No	No	No
Heart	No	No	No
Liver	No	No	No
Placenta	17 alignments*	No	No
Skeletal muscle	No	No	No
Skin	No	No	No
Testis	33 alignments*	1 alignment	1 alignment
Thymus	No	No	No
Thyroid	No	No	No
White blood cells	No	No	No
T21 blood cells	No	No	No
Normal control blood cells	No	No	No

^*^The first exon junction (E1-E2), detected in placenta and testis, is the same of the first exon junctions found in *KCNJ6-202* and in the other *DSCR4* isoforms.

**TABLE 6:** Summary results of *DSCR4-203* expression by SRA-BLAST analysis. The results of alignments between the query sequences derived from *DSCR4-203* and the reads generated by RNA-Seq experiments were reported in this table for each tissue. Only alignments with at least 95% of query cover and 97% of identity were considered as significant and reported in the table.

*DSCR4-203*					
Tissue	Expression				
	E1-E2	E2-E3	E3-E4	E4-E5	E5-E6
Adrenal Gland	No	No	No	No	No
Brain	No	No	No	No	No
Cerebellum	No	No	No	No	No
Cerebral cortex	No	No	No	No	No
Heart	No	No	No	No	No
Liver	No	No	No	No	No
Placenta	17 alignments*	No	No	No	No
Skeletal muscle	No	No	No	No	No
Skin	No	No	No	No	No
Testis	33 alignments*	No	No	No	No
Thymus	No	No	No	No	No
Thyroid	No	No	No	No	No
White blood cells	No	No	No	No	No
T21 blood cells	No	No	No	No	No
Normal control blood cells	No	No	No	No	No

^*^The first exon junction (E1-E2), detected in placenta and testis, is the same of the first exon junctions found in *KCNJ6-202* and in the other *DSCR4* isoforms.

#### 3.2.3 Sequence Alignment Between RNA-Seq Experiments and *DSCR4-201* Transcript

The reads generated by RNA-Seq experiments were aligned with exon junctions of *DSCR4-201* transcript. The alignments predict the expression of the entire transcript in placenta and testis, confirming the previous literature data (Saber et al., 2016) (see Table 4). For more details see Supplementary Table S5.

#### 3.2.4 Sequence Alignment Between RNA-Seq Experiments and *DSCR4-202* Transcript

The reads generated by RNA-Seq experiments were aligned with *DSCR4-202* transcript. The alignments predict the expression of the entire *DSCR4-202* transcript only in testis (see Table 5). For more details see Supplementary Table S6.

#### 3.2.5 Sequence Alignment Between RNA-Seq Experiments and *DSCR4-203* Transcript

The reads generated by RNA-Seq experiments were aligned with the exon junctions of *DSCR4-203* transcript and no alignments with the entire isoform were reported (see Table 6). For more details see Supplementary Table S7.

### 3.2 *KCNJ6* and *DSCR4 in vitro* Characterization: RT-PCR and Sanger Sequencing

Reverse Transcription-Polymerase chain reaction (RT-PCR) was used to perform *KCNJ6* and *DSCR4* expression profiles in several tissues (see Supplementary Data Set S1).

**FIGURE 2:**
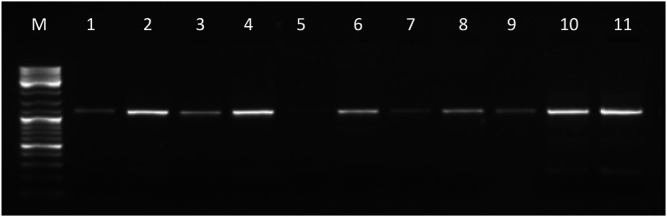
Gel electrophoresis picture of the entire *KCNJ6-201* transcript amplified by primer pairs on E1-E4 (1,167 bp). The marker (lane M) used for the lecture was 1 µL of GeneRuler DNA ladder Mix (ThermoFisher Scientific). Five µL of PCR products were loaded in the following lanes: adrenal gland (lane 1), brain (lane 2), cerebellum (lane 3), cerebral cortex (lane 4), heart (lane 5), placenta (lane 6), skeletal muscle (lane 7), thymus (lane 8), thyroid (lane 9), T21 fibroblast (lane 10), and normal control fibroblast (lane 11).

The entire *KCNJ6-201* transcript, amplified by primer pairs on E1-E4 (see Supplementary Table S2), was detected in adrenal gland, brain, cerebellum, cerebral cortex, heart, placenta, skeletal muscle, skin, thymus, and thyroid (see Figure 2). We obtained part of the transcript in several tissues (for more details see Supplementary Table S8a). Sanger sequencing in brain confirmed PCR results.

**FIGURE 3:**
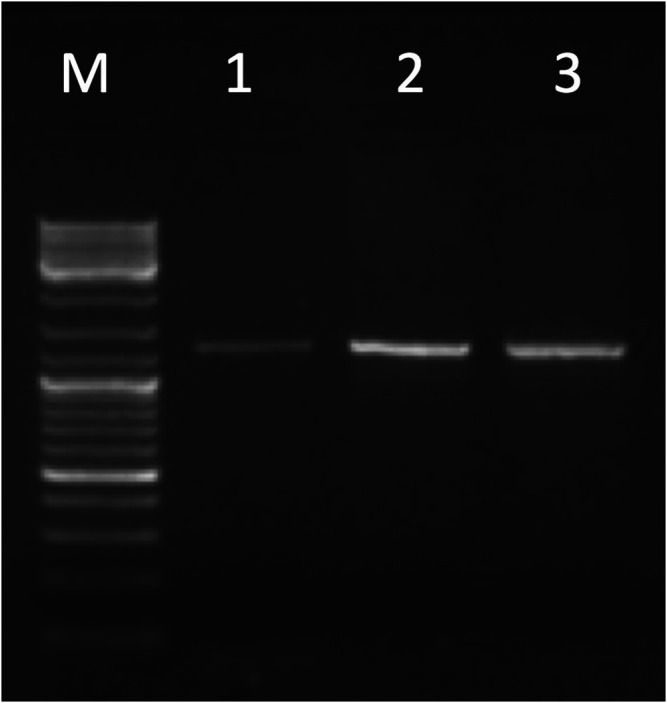
Gel electrophoresis picture of the entire *KCNJ6-202* transcript amplified by primer pairs on E1b-E4 (1,244 bp). The marker (lane M) used for the lecture was 1 µL of GeneRuler DNA ladder Mix (ThermoFisher Scientific). Five µL of PCR products were loaded in the following lanes: brain (lane 1), cerebellum (lane 2), and placenta (lane 3).

The entire *KCNJ6-202* transcript, amplified by primer pairs on E1b-E4 (see Supplementary Table S2), was detected in brain, cerebellum, and placenta (see Figure 3). We obtained part of the transcript in a few tissues (for more details see Supplementary Table S8b). Sanger sequencing in placenta confirmed PCR results. The sequence exactly between primer pair has been deposited in NCBI GenBank under the accession number OK050111 (https://www.ncbi.nlm.nih.gov/nuccore/OK050111).

**FIGURE 4:**
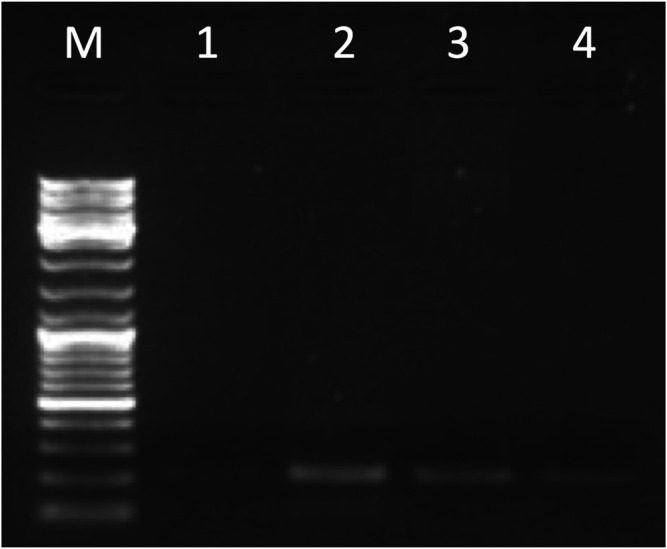
Gel electrophoresis picture of the entire *DSCR4-201* transcript amplified by primer pairs on E1-E3 (215 bp). The marker (lane M) used for the lecture was 1 µL of GeneRuler DNA ladder Mix (ThermoFisher Scientific). Five µL of PCR products were loaded in the following lanes: brain (lane 1), placenta (lane 2), testis (lane 3), and thyroid (lane 4).

The entire *DSCR4-201* transcript, amplified by primer pairs on E1-E3 (see Supplementary Table S2), was detected in brain, placenta, testis, and thyroid (Figure 4; Supplementary Table S8c). Sanger sequencing in thyroid confirmed PCR results.

**FIGURE 5:**
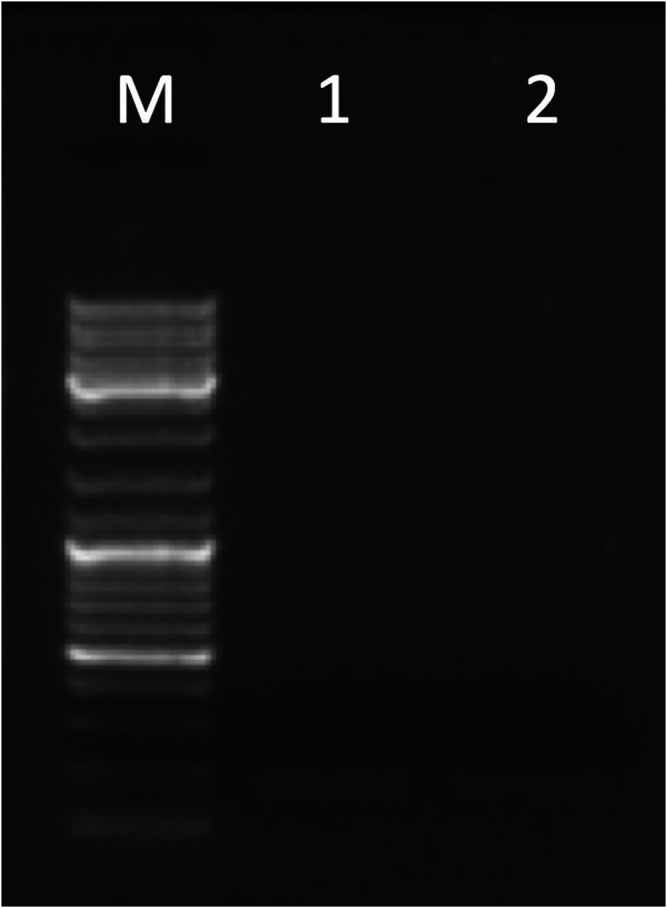
Gel electrophoresis picture of the partial *DSCR4-202* transcript amplified by primer pairs on E3-E4 (177 bp). The marker (lane M) used for the lecture was 1 µL of GeneRuler DNA ladder Mix (ThermoFisher Scientific). Five µL of PCR products were loaded in the following lanes: placenta (lane 1) and testis (lane 2).

The entire *DSCR4-202* transcript was not detected in any tissues (see Supplementary Table S8d), but was detected the expression of E3-E4 (located in the HR-DSCR) in placenta and testis confirmed by Sanger sequencing in both tissues (see Figure 5). The sequence exactly between primer pair has been deposited in NCBI GenBank under the accession number OK318455 (https://www.ncbi.nlm.nih.gov/nuccore/OK318455).

The entire or partial *DSCR4-203* transcript was not detected in any tissues (see Supplementary Table S8e).

## 4 Discussion

HR-DSCR is the minimal region of Hsa21 shared by all subjects with PT21 and a confirmed diagnosis of DS. It is described as an intergenic region, but its link with the typical DS symptoms suggests that it could contain active *loci*. In this work, starting from HR-DSCR limits designed by Pelleri and coll. (Pelleri et al., 2016), Ensembl Genome Browser was consulted in order to investigate the presence of transcripts annotated in this region.

*KCNJ6-201* and *DSCR4-201* are validated transcripts and are deposited on the NCBI Gene database, even if the role of *DSCR4-201* is not yet well characterized. In this work, the computational and molecular analyses confirmed the gene expression profile reported in NCBI Gene database and through an enlarged panel of tissues we enriched the knowledge on their tissue specificity (see Figures 2, 4).

For the first time we performed the *in vitro* characterization of *KCNJ6-202, DSCR4-202,* and *DSCR4-203* transcripts, automatically annotated in Ensembl Genome Browser from massive RNA-Seq data.

The entire *KCNJ6-202* transcript was detected in brain, cerebellum, and placenta, while the entire *DSCR4-202* and *DSCR4-203* transcripts were not detected in any tissues. In particular, a 177 bp amplicon was detected using a primer pair based on exon 3 and exon 4 of *DSCR4-202* that was not connected with its previous exons. *DSCR4-203* was not detected by either computational and molecular analyses.

All the transcripts were visible on agarose gel after 45 cycles of PCR and only in specific tissues. These characteristics are typical of lncRNA, which follow a specific timing and tissue expression pattern, and have a key role in cell differentiation, organogenesis, and tissue homeostasis (Schmitz et al., 2016).

LncRNAs can interact with DNA and RNA molecules, transcription factors, and they participate in various biological processes such as DNA methylation, histone modification, chromatin remodeling, up-regulation or down-regulation of target genes (Li et al., 2016; Kaikkonen and Adelman, 2018). All *DSCR4* isoforms are annotated as lncRNA in Ensembl database and HR-DSCR could contain regulatory RNA elements in its genomic *locus*. Instead, *KCNJ6-202* function is associated to *KCNJ6-201* because it is thought that they code for the same protein.

Interestingly, *DSCR4-202* has the third and fourth exons in the HR-DSCR (see Figure 1), while *KCNJ6-202* includes HR-DSCR in a long intron between its second and third exon (see Figure 1). 9ecent studies on intronic sequences had detected a new class of non-coding RNA (ncRNA), defined as stable intronic sequence RNA (sisRNAs), which are not rapidly degraded and could play a regulatory role as lncRNA (Chan and Pek, 2019). Thus, the HR-DSCR region should no longer be considered a silent region because it is part of an active *locus* where splicing occurs.

The organization of HR-DSCR is extremely complex and was conserved during the evolution of the *Hominidae* family, highlighting a role in the development of higher brain function (Saber et al., 2016). Recently, wild-type *DSCR4* was overexpressed in human non-cancerous cells and differential gene expression analysis was performed to measure the consequences on the cell transcriptome. The results suggested that *DSCR4* could have a role in the regulation of interconnected biological pathways related to cell migration, coagulation and immune system consistent with well-known pathways affected in subjects with DS (Saber et al., 2021) and confirming the complexity of its gene function.

In order to understand the function of this region it is necessary to define the function of the transcripts included in it. Further studies are necessary to characterize the organization of the HR-DSCR *locus* and of the *KCNJ6-202* and *DSCR4-202* transcripts that intersect it and to clarify their properties and function in the tissues analyzed*.*

Due to the particular structure of the HR-DSCR, which is unique to the *Hominidae* family, we suggest avoiding the use of mouse as a model organism to test the effects of overexpression of the transcripts crossing the HR-DSCR. Actually, it may be difficult to make assumptions about complex *loci* across different species. This topic is exemplified by another gene located on Hsa21 named *DSCAM* which can produce only a few splicing isoforms in humans, while it has the potential to produce 38,016 isoforms in *Drosophila melanogaster*, contributing to the formation of complex patterns of neuronal connections (Wojtowicz et al., 2004). Aside from the considerations regarding conservation of function of complex *loci* across different species, other limitations for the use of mouse as a model organism for DS should be considered (Pelleri et al., 2016; Strippoli et al., 2019), such as differences in the content of homologous genes between trisomy 21 in humans and trisomy of mouse chromosome 16 in the case of the most used murine strain (Ts65Dn) (Davisson et al., 1990) and more generally the lack of superior cognitive functions like language and abstract thinking in mice that are the main markers of ID in DS.

In conclusion, this study confirms the importance of a systematic and thorough analysis of apparently silent regions of the human genome. In particular, the strict association between HR-DSCR and diagnosis of DS deriving from clinical data suggests the need for an intensive study of this region to understand the genotype-phenotype relationship in DS and to identify targets for a rational treatment of the ID associated to DS.

## Data Availability

The original contributions presented in the study are publicly available. These data can be found here: National Center for Biotechnology Information (NCBI) GenBank database under accession number OK050111 and OK318455.

## References

[B1] Antonaros F. Lanfranchi S. Locatelli C. Martelli A. Olivucci G. Cicchini E. (2021a). One-carbon Pathway and Cognitive Skills in Children with Down Syndrome. Sci. Rep. 11, 4225. 10.1038/s41598-021-83379-7 33608632 PMC7895965

[B2] Antonaros F. Zenatelli R. Guerri G. Bertelli M. Locatelli C. Vione B. (2021b). The Transcriptome Profile of Human Trisomy 21 Blood Cells. Hum. Genomics 15, 25. 10.1186/s40246-021-00325-4 33933170 PMC8088681

[B3] Caspersson T. Zech L. Johansson C. Modest E. J. (1970). Identification of Human Chromosomes by DNA-Binding Fluorescent Agents. Chromosoma 30, 215–227. 10.1007/BF00282002 4193398

[B4] Chan S. N. Pek J. W. (2019). Stable Intronic Sequence RNAs (sisRNAs): An Expanding Universe. Trends Biochem. Sci. 44, 258–272. 10.1016/j.tibs.2018.09.016 30391089

[B5] Chomczynski P. Sacchi N. (1987). Single-step Method of RNA Isolation by Acid Guanidinium Thiocyanate-Phenol-Chloroform Extraction. Anal. Biochem. 162, 156–159. 10.1016/0003-2697(87)90021-2 2440339

[B6] Cooper A. Grigoryan G. Guy-David L. Tsoory M. M. Chen A. Reuveny E. (2012). Trisomy of the G Protein-Coupled K+ Channel Gene, Kcnj6, Affects Reward Mechanisms, Cognitive Functions, and Synaptic Plasticity in Mice. Proc. Natl. Acad. Sci. 109, 2642–2647. 10.1073/pnas.1109099109 22308328 PMC3289362

[B7] Daniel A. (1979). Normal Phenotype and Partial Trisomy for the G Positive Region of Chromosome 21. J. Med. Genet. 16, 227–229. 10.1136/jmg.16.3.227 157396 PMC1012698

[B8] Davisson M. T. Schmidt C. Akeson E. C. (1990). Segmental Trisomy of Murine Chromosome 16: a New Model System for Studying Down Syndrome. Prog. Clin. Biol. Res. 360, 263–280. 2147289

[B9] Delabar J.-M. Theophile D. Rahmani Z. Chettouh Z. Blouin J.-L. Prieur M. (1993). Molecular Mapping of Twenty-Four Features of Down Syndrome on Chromosome 21. Eur. J. Hum. Genet. 1, 114–124. 10.1159/000472398 8055322

[B10] Dunn C. A. Romanish M. T. Gutierrez L. E. Van De Lagemaat L. N. Mager D. L. (2006). Transcription of Two Human Genes from a Bidirectional Endogenous Retrovirus Promoter. Gene 366, 335–342. 10.1016/j.gene.2005.09.003 16288839

[B11] Eggermann T. Schwanitz G. (2011). “Genetics of Down Syndrome,” in Genetics and Etiology of Down Syndrome. Editor Dey S. (Croatia: InTech), 3–22. 10.5772/17817

[B12] Engels W. R. (1993). Contributing Software to the Internet: the Amplify Program. Trends Biochem. Sci. 18, 448–450. 10.1016/0968-0004(93)90148-g 8291093

[B13] Fagerberg L. Hallström B. M. Oksvold P. Kampf C. Djureinovic D. Odeberg J. (2014). Analysis of the Human Tissue-specific Expression by Genome-wide Integration of Transcriptomics and Antibody-Based Proteomics. Mol. Cell Proteomics 13, 397–406. 10.1074/mcp.m113.035600 24309898 PMC3916642

[B14] Gustavson K. H. (1964). Down's Syndrome: A Clinical and Cytogenetic Investigation. Uppsala: Almqvist & Wiksell.

[B15] Hamerton J. (1971). Banding Patterns of Metaphase Chromosomes in Down's Syndrome. The Lancet 298, 709. 10.1016/s0140-6736(71)92281-1 4105741

[B16] Hattori M. Fujiyama A. Taylor T. D. Watanabe H. Yada T. Park H.-S. (2000). The DNA Sequence of Human Chromosome 21. Nature 405, 311–319. 10.1038/35012518 10830953

[B17] Kaikkonen M. U. Adelman K. (2018). Emerging Roles of Non-coding RNA Transcription. Trends Biochem. Sci. 43, 654–667. 10.1016/j.tibs.2018.06.002 30145998

[B18] Korbel J. O. Tirosh-Wagner T. Urban A. E. Chen X.-N. Kasowski M. Dai L. (2009). The Genetic Architecture of Down Syndrome Phenotypes Revealed by High-Resolution Analysis of Human Segmental Trisomies. Proc. Natl. Acad. Sci. 106, 12031–12036. 10.1073/pnas.0813248106 19597142 PMC2709665

[B19] Lejeune J. Gautier M. Turpin R. (1959). Study of Somatic Chromosomes from 9 Mongoloid Children. C R. Hebd Seances Acad. Sci. 248, 1721–1722. 13639368

[B20] Li R. Zhu H. Luo Y. (2016). Understanding the Functions of Long Non-coding RNAs through Their Higher-Order Structures. Int. J. Mol. Sci. 17. 10.3390/ijms17050702 PMC4881525 27196897

[B21] Lyle R. Béna F. Gagos S. Gehrig C. Lopez G. Schinzel A. (2009). Genotype-phenotype Correlations in Down Syndrome Identified by Array CGH in 30 Cases of Partial Trisomy and Partial Monosomy Chromosome 21. Eur. J. Hum. Genet. 17, 454–466. 10.1038/ejhg.2008.214 19002211 PMC2986205

[B22] Mccormick M. Schinzel A. Petersen M. B. Stetten G. Driscoll D. J. Cantu E. S. (1989). Molecular Genetic Approach to the Characterization of the ?Down Syndrome Region? of Chromosome 21. Genomics 5, 325–331. 10.1016/0888-7543(89)90065-7 2529205

[B23] Nakamura A. Hattori M. Sakaki Y. (1997). A Novel Gene Isolated from Human Placenta Located in Down Syndrome Critical Region on Chromosome 21. DNA Res. 4, 321–324. 10.1093/dnares/4.5.321 9455479

[B24] Niebuhr E. (1974). Down's Syndrome. Hum. Genet. 21, 99–101. 10.1007/bf00278575 4276065

[B25] Pelleri M. C. Cicchini E. Locatelli C. Vitale L. Caracausi M. Piovesan A. (2016). Systematic Reanalysis of Partial Trisomy 21 Cases with or without Down Syndrome Suggests a Small Region on 21q22.13 as Critical to the Phenotype. Hum. Mol. Genet. 25, 2525–2538. 10.1093/hmg/ddw116 27106104 PMC5181629

[B26] Pelleri M. C. Cicchini E. Petersen M. B. Tranebjaerg L. Mattina T. Magini P. (2019). Partial Trisomy 21 Map: Ten Cases Further Supporting the Highly Restricted Down Syndrome Critical Region (HR-DSCR) on Human Chromosome 21. Mol. Genet. Genomic Med. 7, e797. 10.1002/mgg3.797 31237416 PMC6687668

[B27] Pelleri M. C. Gennari E. Locatelli C. Piovesan A. Caracausi M. Antonaros F. (2017). Genotype-phenotype Correlation for Congenital Heart Disease in Down Syndrome through Analysis of Partial Trisomy 21 Cases. Genomics. 10.1016/j.ygeno.2017.06.004 28648597

[B28] Piovesan A. Antonaros F. Vitale L. Strippoli P. Pelleri M. C. Caracausi M. (2019). Human Protein-Coding Genes and Gene Feature Statistics in 2019. BMC Res. Notes 12, 315. 10.1186/s13104-019-4343-8 31164174 PMC6549324

[B29] Piovesan A. Caracausi M. Antonaros F. Pelleri M. C. Vitale L. (2016). GeneBase 1.1: a Tool to Summarise Data from NCBI Gene Datasets and its Application to an Update of Human Gene Statistics. Database (Oxford) 2016, baw153. 10.1093/database/baw153 28025344 PMC5199132

[B30] Rahmani Z. Blouin J. L. Creau-Goldberg N. Watkins P. C. Mattei J. F. Poissonnier M. (1989). Critical Role of the D21S55 Region on Chromosome 21 in the Pathogenesis of Down Syndrome. Proc. Natl. Acad. Sci. 86, 5958–5962. 10.1073/pnas.86.15.5958 2527368 PMC297750

[B31] Saber M. M. Adeyemi Babarinde I. Hettiarachchi N. Saitou N. (2016). Emergence and Evolution of Hominidae-specific Coding and Noncoding Genomic Sequences. Genome Biol. Evol. 8, 2076–2092. 10.1093/gbe/evw132 27289096 PMC4987104

[B32] Saber M. M. Karimiavargani M. Uzawa T. Hettiarachchi N. Hamada M. Ito Y. (2021). Possible Roles for the Hominoid-specific DSCR4 Gene in Human Cells. Genes Genet. Syst. 96, 1–11. 10.1266/ggs.20-00012 33762515

[B33] Schmitz S. U. Grote P. Herrmann B. G. (2016). Mechanisms of Long Noncoding RNA Function in Development and Disease. Cell. Mol. Life Sci. 73, 2491–2509. 10.1007/s00018-016-2174-5 27007508 PMC4894931

[B34] Shapiro B. L. (1999). The Down Syndrome Critical Region. J. Neural Transm. Suppl. 57, 41–60. 10.1007/978-3-7091-6380-1_3 10666667

[B35] Sharrocks A. (1994). “The Design of Primer for PCR,” in PCR Technology—Current Innovations. Editors Griffin H. G. Griffin A. M. (Boca Raton, FL: CRC Press), 5–11.

[B36] Strippoli P. Pelleri M. C. Piovesan A. Caracausi M. Antonaros F. Vitale L. (2019). Genetics and Genomics of Down Syndrome. Int. Rev. Res. Dev. Disabil. 56, 1–39. 10.1016/bs.irrdd.2019.06.001

[B37] Wojtowicz W. M. Flanagan J. J. Millard S. S. Zipursky S. L. Clemens J. C. (2004). Alternative Splicing of Drosophila Dscam Generates Axon Guidance Receptors that Exhibit Isoform-specific Homophilic Binding. Cell 118, 619–633. 10.1016/j.cell.2004.08.021 15339666 PMC2691713

[B38] Ye J. Coulouris G. Zaretskaya I. Cutcutache I. Rozen S. Madden T. L. (2012). Primer-BLAST: a Tool to Design Target-specific Primers for Polymerase Chain Reaction. BMC Bioinformatics 13, 134. 10.1186/1471-2105-13-134 22708584 PMC3412702

